# Pure spin current devices based on ferromagnetic topological insulators

**DOI:** 10.1038/srep36070

**Published:** 2016-10-26

**Authors:** Matthias Götte, Michael Joppe, Thomas Dahm

**Affiliations:** 1 Universität Bielefeld, Fakultät für Physik, Postfach 100131, D-33501 Bielefeld, Germany

## Abstract

Two-dimensional topological insulators possess two counter propagating edge channels with opposite spin direction. Recent experimental progress allowed to create ferromagnetic topological insulators realizing a quantum anomalous Hall (QAH) state. In the QAH state one of the two edge channels disappears due to the strong ferromagnetic exchange field. We investigate heterostructures of topological insulators and ferromagnetic topological insulators by means of numerical transport calculations. We show that spin current flow in such heterostructures can be controlled with high fidelity. Specifically, we propose spintronic devices that are capable of creating, switching and detecting pure spin currents using the same technology. In these devices electrical currents are directly converted into spin currents, allowing a high conversion efficiency. Energy independent transport properties in combination with large bulk gaps in some topological insulator materials may allow operation even at room temperature.

In contrast to charge based electronic devices, spintronic devices are supposed to include or solely use the spin degree of freedom of charge carriers^1^,^2^. This requires materials and methods that allow the creation and control of pure spin currents in solid-state systems. Currently there exist a few methods capable of creating pure spin currents, like spin Hall effect^3^,^4^, spin pumping^5^,^6^,^7^ and spin Seebeck effect^7^,^8^,^9^. The detection of spin currents usually exploits the inverse spin Hall effect^5^,^6^,^7^,^8^,^9^. However, the efficiency of these methods is low regarding the power needed to create a sizeable spin current^7^.

Topological insulators (TIs) are materials, which are insulating in the bulk but possess conducting states at the surface or, in the two-dimensional (2D) limit, at the edges^10^,^11^. The spin of these edge states is locked with the propagation direction along the edge, i.e. electrons with opposite spin orientation move in opposite direction^12^,^13^,^14^,^15^. In addition, the surface states are topologically protected which precludes backscattering and conserves the spin-momentum locking of the edge states^16^. These properties make TIs promising candidates for spintronic devices^17^,^18^.

Recent experimental progress has resulted in creation of ferromagnetic topological insulators. Ferromagnetism is either induced by doping with transition metal atoms^19^,^20^,^21^,^22^,^23^,^24^ or by the proximity effect^25^,^26^,^27^,^28^,^29^,^30^. When the ferromagnetic exchange field (FEF) is directed perpendicular to the 2D TI sheet the edge state dispersion remains robust^31^,^32^ and does not acquire a gap. As has been pointed out by Liu *et al*.^33^, a transition into a quantum anomalous Hall state (QAH) occurs when the FEF exceeds a critical strength. Then, one pair of edge states is pushed into the bulk and disappears. In the QAH state only a single spin direction can propagate along the edge in a single direction, similar as in the quantum Hall state^33^,^34^. The QAH state has been demonstrated experimentally in several ferromagnetic topological insulators^21^,^22^,^23^,^24^. Such ferromagnetic TIs thus allow to switch and selectively turn off certain edge state channels by changing the magnetization direction. Because of a large bulk gap in some TI materials^35^, e.g. about 0.3 eV in Bi_2_Se_3_^36^,^37^ and even larger gaps in newly predicted 2D materials^38^,^39^, devices based on these materials could even operate at room temperature^28^.

Previous experiments and proposals for TI spintronic devices mainly focus on the injection of spin or spin polarized electrons into the surface states of TIs^40^,^41^,^42^,^43^ or on the manipulation of spin polarized currents^44^,^45^,^46^,^47^. It is only very recently that pure spin current devices have been considered^48^.

In the present work, we demonstrate by numerical transport calculations that heterostructures combining topological insulators and ferromagnetic topological insulators can steer spin currents with high fidelity in a controlled way. We present devices that allow the creation, switching and detection of pure spin currents, all using the same type of heterostructures. An electrical voltage is directly converted into a pure spin current, which allows a higher conversion efficiency than the above mentioned known methods for spin current generation and detection. Our proposals are supported by numerical time evolution of wave packets on finite 2D lattices. We are using a model and parameters suitable for Bi_2_Se_3_ thin films (see methods section), however our proposals can be applied to other materials as well.

## Results

### Gapless states

**Figure 1:**
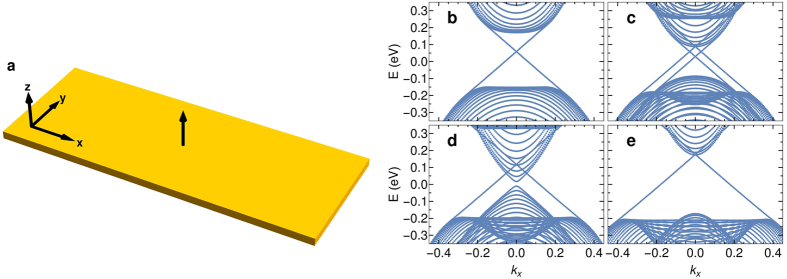
Dispersion. Numerical dispersion of bulk and edge states of a TI bounded by vacuum in *y*-direction as shown in (**a**). Different strengths *V*_*z*_ of an FEF applied in *z*-direction are shown. (**b**) *V*_*z*_ = 0. Edge states are twofold degenerate, with counterpropagating spin-up and spin-down states at each edge. (**c**) *V*_*z*_ = 0.5*M*_2*D*_, with *M*_2*D*_ = 0.17 eV (see methods section). Spin-up and spin-down edge states split. The bulk gap becomes smaller. (**d**) *V*_*z*_ = *M*_2*D*_. Spin-down edge states are completely removed and the bulk gap is nearly closed. (**e**) *V*_*z*_ = 2*M*_2*D*_. The bulk gap has reopened to approximately the same energy range as in (**b**). Only non-degenerate spin-up edge states are present. The system is now in a quantum anomalous Hall state.

The goal of this work is to construct spintronic devices from TIs by using ferromagnetic exchange fields (FEFs). Before we examine the above mentioned heterostructures, we first need to understand how the gapless edge states behave in the presence of FEFs. For that purpose we first review the transition of the Quantum Spin Hall (QSH) state into a QAH state with increasing FEF following ref. 31. We have calculated the dispersion of edge and bulk states by exact diagonalization of a lattice model for a thin ferromagnetic Bi_2_Se_3_ strip as detailed in the methods section below. Results are shown in Fig. 1 and discussed in the following for different strengths *V*_*z*_ of an FEF applied perpendicular to the surface plane.

In a pure 2D TI, gapless states exist only at the edges of the TI where it is in contact to an ordinary insulator or vacuum (Fig. 1b). They are twofold degenerate at each edge (fourfold degenerate, considering both edges as in Fig. 1). Their spin polarization is directed into *z*-direction (perpendicular to the surface plane of the 2D sheet) and they possess an approximately linear dispersion. Note, however that in general each edge state is a superposition of different orbital states with different spin components, which partially compensate each other. Therefore it is more appropriate to speak of two orthogonal pseudo-spin states rather than spin-states. Whereas the absolute value of the spin polarization of both states is the same, one of them is dominated by spin-up and propagates clockwise along the edges while the other one is dominated by spin-down and propagates counterclockwise. In the following we will refer to the first of these pseudo-spin states as spin-up and to the second ones as spin-down and indicate them in any figure by green and red color, respectively.

An FEF in *z*-direction lifts the degeneracy of the edge states without opening a gap (Fig. 1c–e)^49^. For small FEF, the edge state dispersions are shifted in momentum (Fig. 1c). When the FEF becomes of the order of half the bulk gap, two of the bulk subbands touch. Then the bulk gap is closed and one pair of edge states is absorbed into the bulk (Fig. 1d). When the FEF becomes larger than half of the initial bulk gap, the bulk gap reopens which leaves only a single pair of edge states. If the field is strong enough (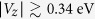 in our case, see Fig. 1e), one pair of edge states is completely removed and the bulk gap is restored. The system is then in a quantum anomalous Hall state^31^,^32^. The remaining states are only shifted in momentum and have a slightly modified group velocity. The degree of spin polarization remains unchanged, however. Which states remain inside the bulk gap depends on the sign of the FEF, in which a positive (negative) FEF removes spin-down (up) states. This analysis of the influence of an FEF on the edge states shows that one can selectively remove one pair of edge states by applying a moderate exchange field of the order of 0.3 eV. In the following we will refer to this situation of a TI with a sufficiently strong FEF as ferromagnetic topological insulator (FTI).

As has been pointed out by Liu *et al*.^33^, the QSH state in the absence of the FEF can be seen as two copies of QAH states with opposite Hall conductivities, such that the total Hall conductivity becomes zero^50^. When the critical value of the FEF is reached, one of these two copies experiences a closure of the bulk gap and its Hall conductivity becomes zero. Then the remaining copy carries a nonzero Hall conductivity and the whole system becomes a QAH insulator^51^.

**Figure 2:**
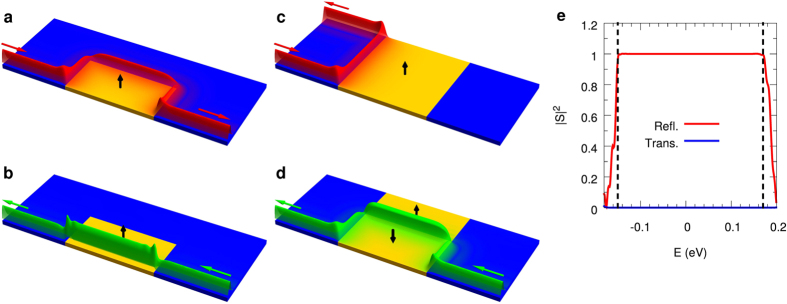
Propagation of gapless states at local FEFs. (**a**–**d**) Local density of states at different local FEFs (orange; black arrows indicate the polarization) on a narrow TI strip (blue). Green color indicates spin-up states and red color spin-down states. The corresponding red and green arrows show the entrance and exit of an electron in that state. At the lower edge, spin-up states come from the right and spin-down states from the left. Fermi energy is chosen as *E* = 0, approximately in the center of the bulk gap. (**e**) Energy dependent reflection and transmission probabilities corresponding to **c**. Reflection is nearly perfect over the largest part of the TI bulk gap. The gap edges are shown by the vertical dashed lines. Setups (**a**,**b**,**d**) show perfect transmission.

As a next step, we consider heterostructures of a TI without FEF and an FTI as shown in Fig. 2. In order to determine the behavior of the edge states in such an inhomogeneous situation, we have done numerical quantum transport calculations^44^ as detailed in the methods section. We prepare initial electron wave packets in different edge channels and follow their evolution through the system by solving the time dependent Schroedinger equation. The path an electron takes is shown in red color for spin-down states and in green color for spin-up states. Our results show that the presence of an FTI area in a TI does not destroy the pair of gapless states discussed above, but instead pushes them away from the TI edge towards the interface of the TI with the FTI area. For an FTI with positive polarization a spin-down electron coming from the left cannot propagate inside the FTI area and thus takes a detour around the FTI area and then moves on at the same edge of the TI (Fig. 2a). On the other hand, a spin-up electron coming from the right stays at the edge and moves straight through the FTI (Fig. 2b). Changing the polarization of the FTI interchanges the paths of the counterpropagating electrons, i.e. the electron from the right takes the detour and the one from the left moves straight through. When the FTI area is extended to the opposite edge of the TI, as shown in Fig. 2c, one of the spin states is removed at both edges. I.e. for an FTI with positive polarization, an incoming spin-down electron can no longer pass from left to right and vice versa. As the FEF in *z*-direction conserves the pseudo-spin of the electron, it cannot be reflected back at the same edge but instead moves along the FTI-TI interface towards the other TI edge where it propagates back. On the other hand an incoming spin-up electron starting at the upper left corner can propagate at the upper edge right through the FTI area. In this way the structure shown in Fig. 2c can be used to selectively block one of the two edge channels from propagating from left to right. The behavior at the interface of two FTIs with opposite polarization (Fig. 2d) is qualitatively the same as that of an FTI with a TI. As a result, at such an interface both spin states propagate into the same direction. This is consistent with the existence of chiral fermion modes at magnetic domain walls on the surface of 3D TIs^17^.

Depending on the direction of the FEF at the edges of an FTI only gapless states with either clockwise or counterclockwise propagation direction can exist. The spin direction on a given edge depends on whether the interface is with a TI/FTI or with an ordinary insulator.

Local densities of states shown in Fig. 2a–d were calculated for a Fermi energy *E*_*F*_ = 0 in the center of the bulk gap. In Fig. 2e the energy dependence of the transmission and reflection probability of an incoming electron for the situation in Fig. 2c is shown. Here, the blue line shows the probability that an incoming spin-down electron from the lower left corner edge state is transmitted towards the lower right corner edge state. The red line shows the probability that the electron is reflected back into the upper left corner edge state. Here, reflection is perfect for energies within the bulk gap. Analogously, for the situations in Fig. 2a,b, and d transmission is perfect (not shown). For energies outside the bulk gap the transmission and reflection probabilities drop quickly, because an incoming electron is scattered into bulk states then and it becomes unlikely that it ends up in one of the edge states. (Please see the supplementary material for more details on the definition and calculation of the scattering probabilities).

### Devices

The basic concept of all devices discussed in the following is the band structure modification of thin film TIs by local FEFs. These could either be induced by ferromagnetic materials (FM) on top of the TI via proximity effect or by doping with magnetic atoms. Using the results for the propagation channels at different interfaces shown in Fig. 2, we can now construct useful spintronic devices.

In our simulations the FTI areas have an edge length of 64–128 atoms corresponding to 26.5–53 nm. However, the device structures presented in the following in principle could be constructed on an even smaller scale, because the functioning of the devices in only limited by the spatial extent of the gapless edge states, which can be of the order of 1 nm for large band gap materials^35^.

#### Spin current generator

**Figure 3:**
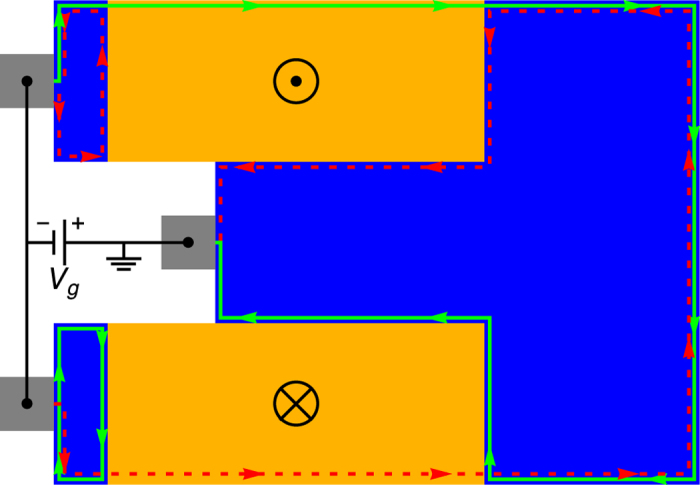
Spin current generator. The spin current generator consists of two injecting and one extracting metallic electrodes (gray) attached to a TI sheet (blue) with an applied voltage *V*_*g*_. Two FTI layers (orange) of opposite polarity prevent direct current flow from the negative to the positive electrode, driving spin currents along the right edge. Current flow in the two spin states is indicated by green solid and red dashed arrows, respectively. The black arrows represent the *z*-polarization of the FM layers (up for positive, down for negative). The local density of states obtained by our quantum transport calculations for this device is shown in Fig. S3 in the supplementary material.

The first device is a spin current generator that creates pure spin currents in a TI. Due to the locking of spin and propagation direction, any charge transport in edge states results in a net spin transport along the edge. To obtain a pure spin current without any net charge transport we have to drive currents of equal magnitude in both directions along the edge. This can be realized by the device shown in Fig. 3. Applying a voltage *V*_*g*_ between the inner positive and the two outer negative metallic contacts (gray), electrons will flow from the negative contacts to the positive contact. We place two FTI areas (orange) of opposite, fixed polarity as illustrated in Fig. 3. As discussed above, only electrons with one polarity can pass the FTI area while the others are reflected. When the upper FTI is polarized in positive *z*-direction, only those electrons with mainly spin-up can pass and must propagate along the right edge, because the other propagation direction is forbidden. The same holds for spin-down electrons at the lower FTI with opposite polarity, resulting in a net spin transport without a net charge transport along the right edge.

In principle, the small TI area (blue) between the metallic electrodes and the FTI is not necessary for the functioning of the device. However, as the FM layer should not overlap with the metallic contact, it should be easier to prepare the device including this area.

We note that in this device the charge current is fully converted into a spin current in principle, as two electron charges 2*e* flowing from the two outer metallic contacts to the inner contact are converted into a total spin flow of *pħ* along the right edge. Here, 0 ≤ *p* ≤ 1 is the spin polarization of the edge states. Reported values for *p* typically range between 0.3 and 0.9 depending on material (see ref. 43 and references therein). In the present model we have *p* = *D*/*B* ≈ 0.35, because the edge states have opposite spin for the two considered orbitals, partially compensating each other. The conversion efficiency is given by 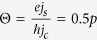, where *j*_*c*_ is the charge and *j*_*s*_ the spin current density. This can be compared with the conversion efficiency of the spin Hall effect as quantified by the spin Hall angle Θ_*SH*_. The largest known spin Hall angles are currently of the order of 0.1^4^,^52^.

The conversion efficiency of the present device depends on temperature as thermal excitation of edge state electrons into bulk states can appear. This thermal effect can be kept small, if the Fermi energy is arranged in the center of the bulk gap and materials with large bulk gaps are chosen. For Bi_2_Se_3_ considered here the bulk gap of 0.3 eV will allow operation of the device at room temperature with high conversion efficiency.

We want to point out that spin-flip scattering at the edges, that could be caused by magnetic impurities or by the Rashba effect due to the coupling to a substrate for example, is not going to affect the conversion efficiency of the device. First of all in the FTI areas spin-flip scattering is forbidden because the spin-up and spin-down edge channels are spatially separated. Along the right edge spin-flip scattering is possible and will affect the resistance of the device, if the length of the edge becomes larger than the spin-flip mean free path, which in topological edge states was reported to be of the order of 2 μm^53^,^54^. However, the conversion efficiency is not changed by spin-flip scattering: consider a spin-flip scattering site at the right edge. If the scattering rate for spin-up electrons scattered backwards into the spin-down channel is *γ*, the scattering rate for spin-down electrons scattered backwards into the spin-up channel will be the same due to reciprocity. Thus, the loss in one channel due to backward spin-flip scattering will be fully compensated for by the reverse process. As a result, both spin current components will remain unchanged by spin-flip scattering and the ratio between spin and charge current will stay the same. (The voltage *V*_*g*_ necessary to drive these currents will increase, though).

#### Spin current detector

**Figure 4:**
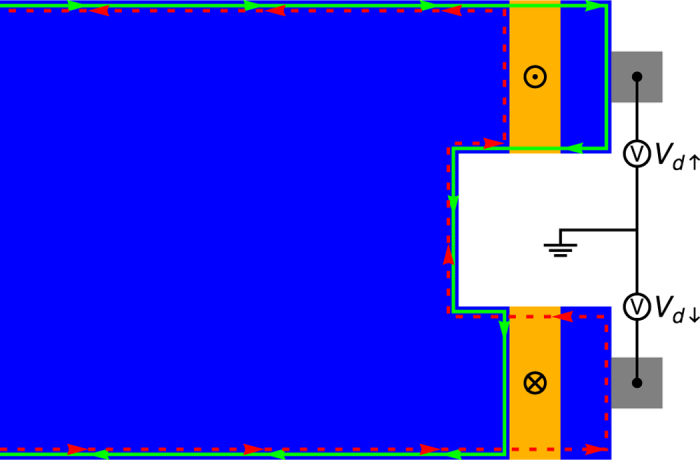
Spin current detector. The spin current detector is basically an inverted generator which consists of two FTI layers. This allows separate measurement of the two spin polarized currents by blocking one of them while the other one can pass. The resulting electrical currents can be measured as voltage drops *V*_*d*↑_ and *V*_*d*↓_ with respect to the common ground with the generator. In the case of a pure spin current, these voltages are equal. The local density of states obtained by our quantum transport calculations for this device is shown in Fig. S5 in the supplementary material.

Because there is no instrument for direct measurement of spin currents, the spin current needs to be transformed back into a charge current in order to be detected. We do this by splitting the spin current into its two counterpropagating parts and measure them individually. This can be achieved by an inversion of the spin current generator, as shown in Fig. 4. The splitting is done by two FM layers with opposite polarity. For positive *z*-magnetization, only the spin-up current can pass the FTI and can be detected at the upper electrode as a voltage drop *V*_*d*↑_ with respect to the common ground with the generator. Such kind of voltage drops at the edges of a 2D TI have been observed previously^14^,^55^. Correspondingly, for negative *z*-magnetization, only the spin-down part can pass the FTI and the resulting current can again be measured as a voltage drop *V*_*d*↓_ at the lower electrode. For a pure spin current, the two measured voltages will be equal, i.e. *V*_*d*↑_ = *V*_*d*↓_. With additional information on resistance and spin polarization of the TI, the net transport of spin angular momentum can be calculated from the measured voltage drop *V*_*d*_.

#### Spin transistor

**Figure 5:**
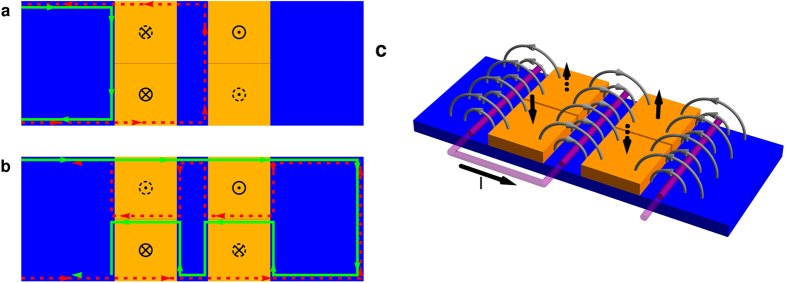
Spin transistor. The spin transistor device consists of four FTI domains from which two are switchable (black dashed arrows). The other two FTI domains are fixed by exchange bias pinning (black solid arrows). (**a**,**b**) Show the two configurations, where (**a**) blocks both spin states while (**b**) allows them to pass. (**c**) Shows a possibility to switch the spin transistor. A current through the wire (purple) induces a magnetic field (gray) with opposite polarity at the two switchable magnetic domains. The loop makes the field stronger and homogeneous. The local density of states obtained by our quantum transport calculations for this device can be found in Fig. S4 in the supplementary material.

A spin transistor is a device that can either reflect or transmit a pure spin current by switching between two configurations. In the first configuration it should reflect all electrons and in the second one it should let them pass, independent of their spin state. A single FTI layer can only block one spin direction while the other one can pass. A spin transistor therefore requires combination of different FTI domains that can be switched individually. The most simple device requires four magnetic domains in two blocks as shown in Fig. 5. While the distance between the two blocks is arbitrary, it is essential that the top and bottom domain of each block are in direct contact in order to form a single domain in the parallel orientation of both domains. In the first configuration (“off”, Fig. 5a), both domains in a block are oriented in parallel, while the orientation of the two blocks is antiparallel. In this way, the left block (negative polarization) reflects the spin-up part and the right block (positive polarization) reflects the spin-down part of the spin current. The second configuration (“on”, Fig. 5b) is reached by switching two of the domains such that the two domains of each block have antiparallel polarization. Here, we assume that both top domains have positive polarization, while both bottom domains have negative polarization. This allows free propagation from left to right along the upper edge of the TI for spin-up electrons and for spin-down electrons along the lower edge. It also opens a channel for the opposite direction at the interface of the two FTI domains for both spin directions.

As the transmission and reflection rates are close to perfect, as was discussed above (Fig. 2e), a high fidelity of this spin transistor at room temperature can be expected, if the Fermi level is chosen near the center of the bulk gap. Spin-flip scattering due to magnetic impurities is possible wherever the two spin channels are at the same location. However, similarly as discussed above for the spin current generator, the scattering rate from the spin-up to the spin-down channel is the same as vice versa due to reciprocity and the fact that there are only two channels available. As a result, the fidelity of this spin transistor is not affected by spin-flip scattering.

In practice, in the “on” configuration shown in Fig. 5b one has to expect a domain wall structure at the edges of the ferromagnetic domains creating a local in-plane magnetization at the position of the edge states. The numerical simulation of such a domain wall structure can be found in the supplementary material. While the in-plane component of the magnetization leads to a modification of the spin and spatial structure of the edge states and creates spin-flip scattering, the total spin current remains unchanged again due to the reciprocity of the two channels.

A possible setup to achieve switching between the two configurations is shown in Fig. 5c. Two out of the four magnetic domains should be fixed (solid arrows). This can be done by exchange bias pinning with an antiferromagnetic material grown on top of the domains^56^. Switching of the other two domains (dashed arrows) could be realized by a current through a wire between the two FTI blocks as illustrated in Fig. 5c. In this configuration the magnetic field of the wire simultaneously switches both domains in opposite directions. However, it may require high currents (of about 0.1 mA–1 mA) to create a sufficiently strong magnetic field, potentially causing heat problems. Alternatively, one could switch the domains separately by a local external field, i.e. a write head. This has the disadvantage of longer switching times. In addition, charge currents along the right edge might appear intermediately, if simultaneous switching is not possible.

As an alternative to the spin transistor device proposed here, one could think of a device that possesses an insulating barrier that can be turned on and off. An idea would be to use an FEF with in-plane polarization, which opens a gap in the edge states^49^. However, the FEF would need to be disengageable for this purpose. In the new class of topological crystalline insulators an electrical field can open a gap in the edge states by breaking the underlying mirror symmetry^57^, which could also potentially be used for the present purpose.

## Discussion

The recent advent of strong ferromagnetic topological insulators allows separate control of the counterpropagating edge states in these materials. We investigated gapless states in 2D TIs with local FEFs. A local FEF perpendicular to the surface plane does not destroy one of the two edge states, but pushes them inside the TI plane towards the edge of the FEF. The edge states with opposite polarization remain basically unchanged, however. This spatial separation of spin states motivates the construction of spintronic devices. Here, we proposed devices that allow the creation, switching and detection of pure spin currents. Large bulk gaps in some TI materials may allow operation at room temperature, because thermal excitation of edge state electrons into the bulk is strongly suppressed by the bulk gap. Note, that larger gaps also need larger exchange fields to drive the material into the QAH state, which might be a challenging requirement. Nevertheless, at low temperature, our devices should be feasible already in existing materials. Direct conversion of electrical currents into spin currents makes our devices more efficient than presently known methods of spin current generation. Eventually, the efficiency depends only on the spin polarization of the TI material. The devices in principle could be miniaturized down to the nanometer scale, as the length scale is determined by the extent of the edge states. The possibility to build all devices on a single TI sheet avoids lattice mismatches at material interfaces, i.e. potential scattering sites.

## Methods

### Model

In this work we investigate a thin film of Bi_2_Se_3_ as a reference material, which belongs to a class of 3D TIs that can be modelled by the two-orbital model Hamiltonian derived by Liu *et al*.^58^. In principle, also other TI materials like HgTe/CdTe quantum wells^10^ and silicene systems^47^ could be used to construct the pure spin current devices we propose here. However, in these systems the QAH state has not been demonstrated experimentally so far. Also the much larger bulk band gap of Bi_2_Se_3_ of about 0.3 eV promises operation of the devices at higher temperatures.

If the film is thin enough, such that the top and bottom surface states are gapped out, it can be reduced to an effective 2D Hamiltonian using the quantum well approximation in perpendicular direction^31^,^59^. In the lattice regularized version for a square lattice from Li *et al*.^60^ the Hamiltonian then reads


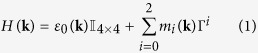
with *ε*_0_(**k**) = *C* + 2*D*(2 − cos*k*_*x*_ − cos*k*_*y*_), *m*_*0*_(**k**) = *M*_2*D*_ − 2*B*(2 − cos*k*_*x*_ − cos*k*_*y*_), *m*_1_(**k**) = *A* sin*k*_*x*_, and *m*_2_(**k**) = *A* sin*k*_*y*_. 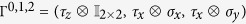 are Dirac Γ matrices in the basis of bonding and antibonding *p*_*z*_ orbitals, where the Pauli matrices *τ*_*i*_ and *σ*_*i*_ operate in orbital and spin space, respectively. Following ref. 31 we set *C* = 0 and use an effective 2D parameter *M*_2*D*_ = 0.17 eV corresponding to a film thickness of 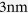. The remaining parameters are derived from Zhang *et al*.^36^ with an effective lattice constant 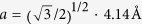: *A* = 1.06 eV, *B* = 3.81 eV and *D* = 1.32 eV. The prefactor 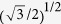 to the real lattice constant given in refs 61, 62, 63 is chosen such that the area of the first Brillouin zone of the square lattice used here equals that of the actual hexagonal lattice.

Local FEFs are modelled by locally adding a Zeeman field of strength *V*_*z*_ in *z*-direction (perpendicular to the surface plane)


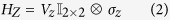


### Eigenstates and wave packets

To calculate the propagation of edge state electrons and obtain their transmission and reflection in our inhomogeneous devices, we use numerical quantum transport calculations in analogy to the work by Krueckl and Richter^44^. This method is a wave packet dynamics calculation in the time domain. In contrast to other methods like the frequently used non-equilibrium Green’s function (NEGF) approach^64^,^65^ no matrix inversions but just matrix multiplications are needed. In addition, the transmission and reflection probabilities for a range of energies is obtained in a single calculational run. This makes the method very efficient when the Hamiltonian is a sparse matrix as in the present case. As a result, much larger systems can be treated.

In order to construct an incoming wave packet for the time evolution, we first consider an infinite strip without FEF. We Fourier transform Equation (1) onto its spatial lattice in the direction perpendicular to the interface, e.g. in *y*-direction. In this case the momentum component parallel to the interface remains a good quantum number and allows calculation of all eigenstates and band structure for discrete momenta *k*_*x*_ by exact numerical diagonalization. Results of such calculations including an FEF are shown in Fig. 1b–e. From these eigenstates, we choose those with energy inside the bulk gap and sort them into four groups (denoted by *v*) selected by the sign of their group velocity 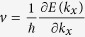 and their spin polarization. The states *χ*_*v*_(*y*, *k*_*x*_) of each group are weighted by a Gaussian distribution


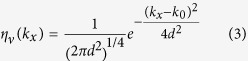


avoiding a sharp momentum cut-off at the ends of the bulk gap. This Gaussian distribution is located around the mean value *k*_0_ of these *k*_*x*_. Then the weighted states are Fourier transformed in *k*_*x*_ to construct wave packets located around a certain starting position *x*_0_


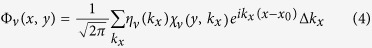


The sum runs over all *k*_*x*_, for which *χ*_*v*_(*y*, *k*_*x*_) is defined, in steps of Δ*k*_*x*_.

### Time evolution

For the time evolution of a wave packet, Equation (1) is Fourier transformed in both directions onto a lattice of size 1024 × 128. Periodic boundary conditions are applied in *x*-direction. Local FTI areas have a size of 128 × 64 or 128 × 128, as illustrated by the orange areas in Fig. 2. The time evolution of a wave packet is then calculated by numerically applying the time evolution operator 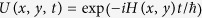 to a starting wave packet initially located close to one of the corners of the lattice.

Following the method of Krueckl and Richter^44^, from the time dependent wave function the energy dependent transmission probabilities


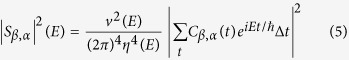


are calculated via Fourier transformation in time of the time dependent overlap *C*_*β*,*α*_(*t*) of the propagating wave packet (denoted by *α*) with four exit wave packets (denoted by *β*) located close to the corners of the lattice. A prefactor removes dependencies on the density of states and the Gaussian weighting factor. The energy dependent quantities *η*(*E*) and the absolute value of the group velocity


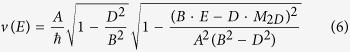


(obtained from the dispersion relation Equation (95) in ref. 32) do not depend on the index *v*. The local densities of states shown in Fig. 2 are proportional to the absolute value of the time-integrated propagating wave packet


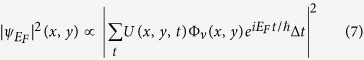


where the factor exp (*iE*_*F*_*t*/*ħ*) allows to select the Fermi energy *E*_*F*_. For more details see Krueckl and Richter^44^ and the corresponding supplementary material.

## Additional Information

**How to cite this article**: Götte, M. *et al*. Pure spin current devices based on ferromagnetic topological insulators. *Sci. Rep.* **6**, 36070; doi: 10.1038/srep36070 (2016).

**Publisher’s note:** Springer Nature remains neutral with regard to jurisdictional claims in published maps and institutional affiliations.

## Supplementary Material

Supplementary Information
