# Supplementary material for: Pure spin current devices based on ferromagnetic topological insulators
Source: Sci Rep. 2016 Oct 26;6:36070. doi: 10.1038/srep36070 (PMC5080548; doi:10.1038/srep36070)
Supplement: Supplementary Information [file srep36070-s1.pdf]

Supplementary material

Pure spin current devices based on ferromagnetic topological insulators

Matthias Götte, Michael Joppe, and Thomas Dahm

Universität Bielefeld, Fakultät für Physik, Postfach 100131, D-33501 Bielefeld, Germany

ABSTRACT

In Ref. 1 we presented quantum transport calculations in heterostructures of topological insulators (TIs) and ferromagnetic TIs (FTIs), i.e. TIs with local ferromagnetic exchange fields. We proposed pure spin current devices based on our calculations. Here, we present some additional results for scattering probabilities, as well as simulations of the proposed devices. In addition, we briefly discuss the effect of domain wall structures on the functioning of the spin transistor.

Scattering probability

The general setup for all quantum transport calculations is shown in Fig. S1. Scattering probabilities $\left|S_{\beta,\alpha}\right|^2(E)$ of the propagating start wave packet Ψ_α into the four possible exit edge state channels are calculated via a Fourier transformation of the time-dependent overlap $C_{\beta,\alpha}(t) = \left\langle \Phi_\beta(x,y) \left| U(x,y,t) \right| \Psi_\alpha(x,y) \right\rangle$ with four exit wave packets Φ_β [1,2]:

$$\left|S_{\beta,\alpha}\right|^2(E) = \frac{v^2(E)}{(2\pi)^4 \eta^4(E)} \left| \sum_t C_{\beta,\alpha}(t)\, e^{iEt/\hbar} \Delta t \right|^2 . \qquad (1)$$

Here, $U(x,y,t) = \exp(-iH(x,y)t/\hbar)$ is the time evolution operator, $v(E)$ the group velocity and $\eta(E)$ the weighting factor of the wave packets (see method section of Ref. 1). We denote the four edge states by $(\downarrow,+)$ for the right-moving spin-down channel, $(\uparrow,+)$ for the right-moving spin-up channel, $(\downarrow,-)$ for the left-moving spin-down channel, and $(\uparrow,-)$ for the left-moving spin-up channel, respectively. The scattering spectrum corresponding to Fig. 2**c+e** of Ref. 1 is shown in Fig. S2 including all edge channels. Inside the bulk gap, electrons are completely reflected by the barrier without spin flip. They are therefore deflected into the left moving spin down channel on the opposite edge. Outside the bulk gap, the number of bulk states, and therefore the probability to scatter into those states, gradually increases. As the quantities $\left|S_{\beta,\alpha}\right|^2(E)$ only take into account scattering into edge states, the probability decreases for energies outside the bulk gap.

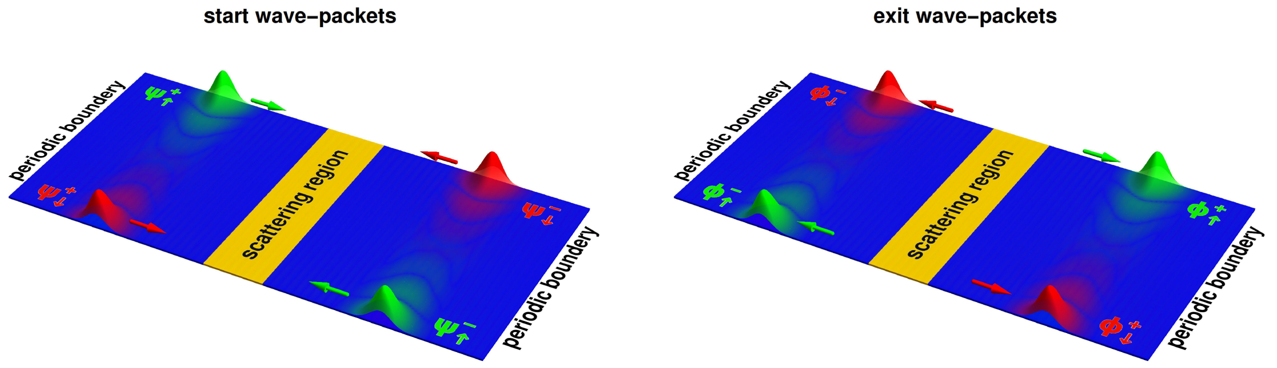

Figure S1. General setup for the quantum transport calculations. During time evolution start wave-packets Ψ_α are scattered inside the scattering region. The probability to find a scattered electron in one of the exit edge state channels, is calculated via the time-dependent overlap with four exit wave packets Φ_β. Exit wave packets are spatially shifted versions of the start wave packets.

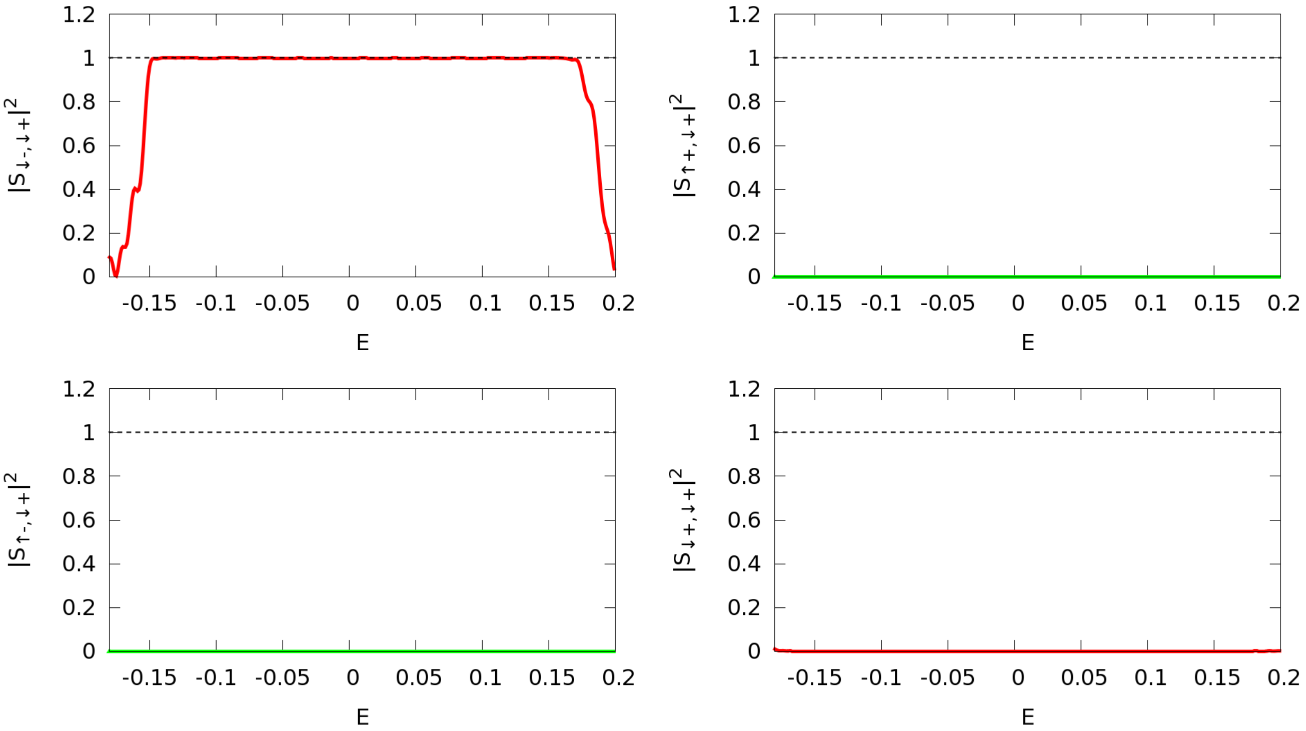

Figure S2. Transmission probabilities corresponding to Fig. 2**c** of Ref. 1, i.e. a barrier with positive exchange field of strength $V_z = 0.34$eV spanning the full width of the scattering region. Inside the bulk gap, electrons are perfectly reflected into the counter propagating channel on the opposite edge with the same spin. Outside the bulk gap the probability gradually drops due to scattering into an increasing number of bulk states.

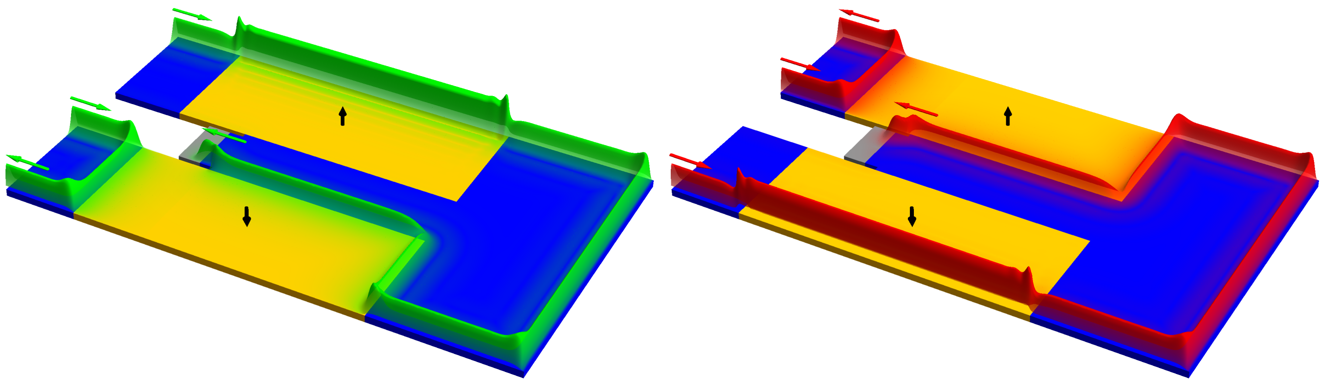

Figure S3. Simulation of the pure spin current generator. Local density of states of spin-up electrons are shown in green on the left and those of spin-down electrons in red on the right. Arrows denote the propagation direction of the wave-packets. In the gray contact area, absorption of the incoming wave-packet is simulated using a small imaginary potential.

Device simulations

In Ref. 1 we proposed pure spin current devices on the basis of simulations for small structures contributing to the devices. In order to get a better picture of the functioning of the full device only sketches were shown. The simulations of the full devices are presented in the following in terms of the local density of states[1,2]

$$\left|\psi_{E_F}\right|^2(x,y) \propto \left|\sum_t U(x,y,t)\,\Phi_\nu(x,y)\,e^{iE_F t/\hbar}\Delta t\right|^2 \tag{2}$$

at the Fermi energy $E_F = 0$ eV. Results are shown separately for the paths of spin-up (green,left) and spin-down (red, right) electrons. FTI areas are denoted by orange color, with black arrows denoting the direction of the ferromagnetic exchange field. TI without exchange field is shown in blue color. Note, that generator and detector have a total width of 256 lattice sides, in contrast to 128 used in the other simulations. This is only done for illustrative purposes. Here, FTI areas have a width of 100 lattice sites.

Domain wall

Our spin transistor device consists of two adjacent ferromagnetic domains, which have opposite magnetization in the "on" configuration. In practice, one has to expect the appearance of a domain wall structure at the interface of these two domains, where the out-of-plane magnetization rotates via an in-plane magnetization. In this case the Zeeman term in the Hamiltonian has to be generalized to

$$H_Z = V_z \mathbb{I}_{2\times 2} \otimes (\mathbf{n}\cdot\boldsymbol{\sigma})\,, \tag{3}$$

with $\mathbf{n}$ being the direction of the exchange field and $\boldsymbol{\sigma} = (\sigma_x, \sigma_y, \sigma_z)$ the vector of Pauli-matrices. The magnetization can rotate either within the domain wall plane (Bloch-type), or perpendicular to the plane (Néel-type). Because Néel-type domain walls are usually dominant in thin films, here, we will focus on a rotation perpendicular to the domain wall plane, i.e. along y-direction. The influence of a Bloch wall is qualitatively the same. For a domain wall of width ξ, located at y_0, the direction of the exchange field can then be written as[3]

$$\mathbf{n}(y) = \left(0, 1/\cosh\frac{(y-y_0)}{\xi}, \tanh\frac{(y-y_0)}{\xi}\right)\,. \tag{4}$$

As has been pointed out by Wakatsuki et al[3] the width of such a domain wall is of the order of a few lattice constants und thus smaller than the extend of the edge states. Following Ref. 3 we use $\xi = 4$, however the results do not change very much as long as ξ remains smaller than the spatial extention of the edge states. Fig. S6 shows the numerical dispersion of an FTI stripe bounded by vacuum in y-direction and with periodic boundary conditions in x-direction, like in Fig. 1**a** of Ref. 1, but now including a domain wall according to Eqs. 3 and 4. There a two twofold degenerate bands crossing the bulk gap. Those with a positive slope are located at the outer boundaries of the FTI and are unaffected by the domain wall. Eigenstates belonging to the other two bands, i.e. those with a negative slope, are located at the domain wall and differ from eigenstates at the edges of a TI/FTI bounded by vacuum. They possess contributions from all orbital and spin components, as shown in the right panel of

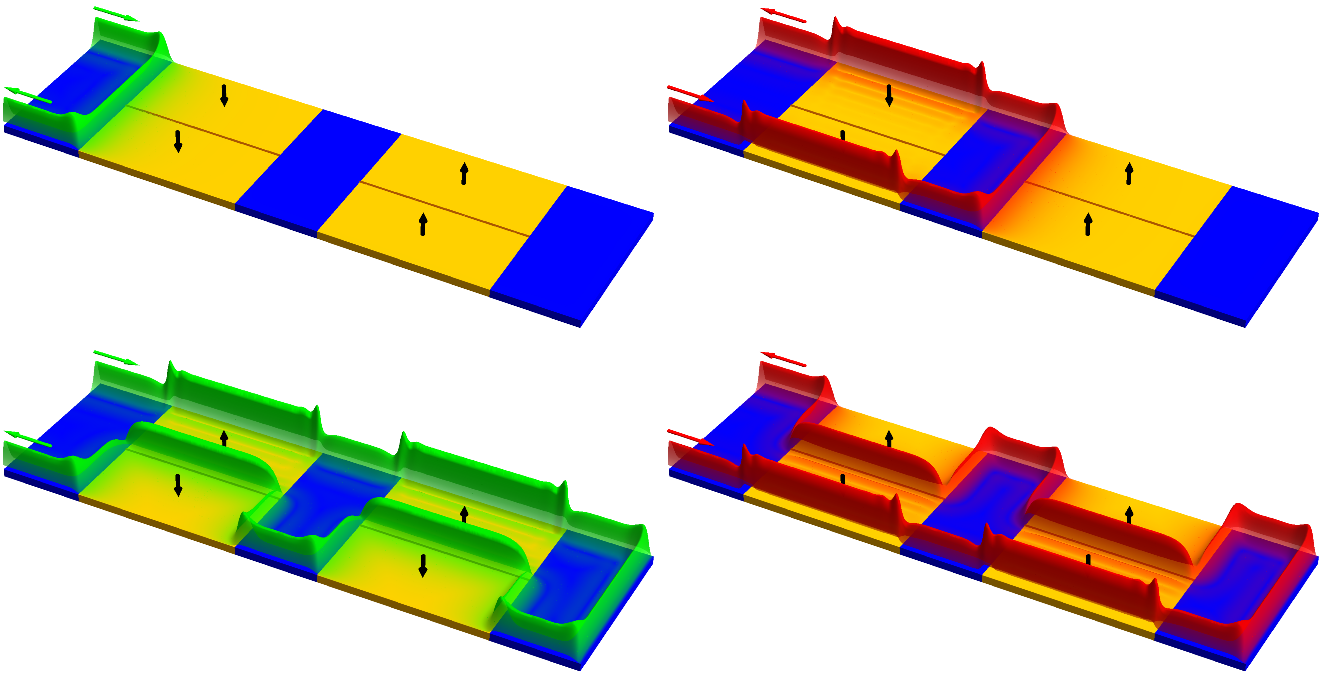

Figure S4. Simulation of the spin current transistor in the "off" (top) and "on" (bottom) configuration. Again, spin-up is shown in green on the left pictures and spin-down in red on the right pictures.

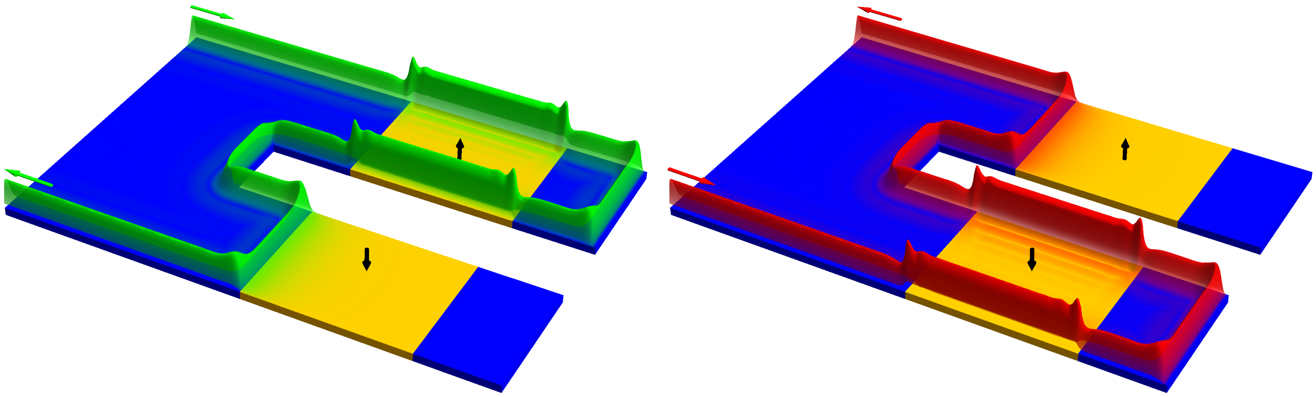

Figure S5. Simulation of the spin current detector.

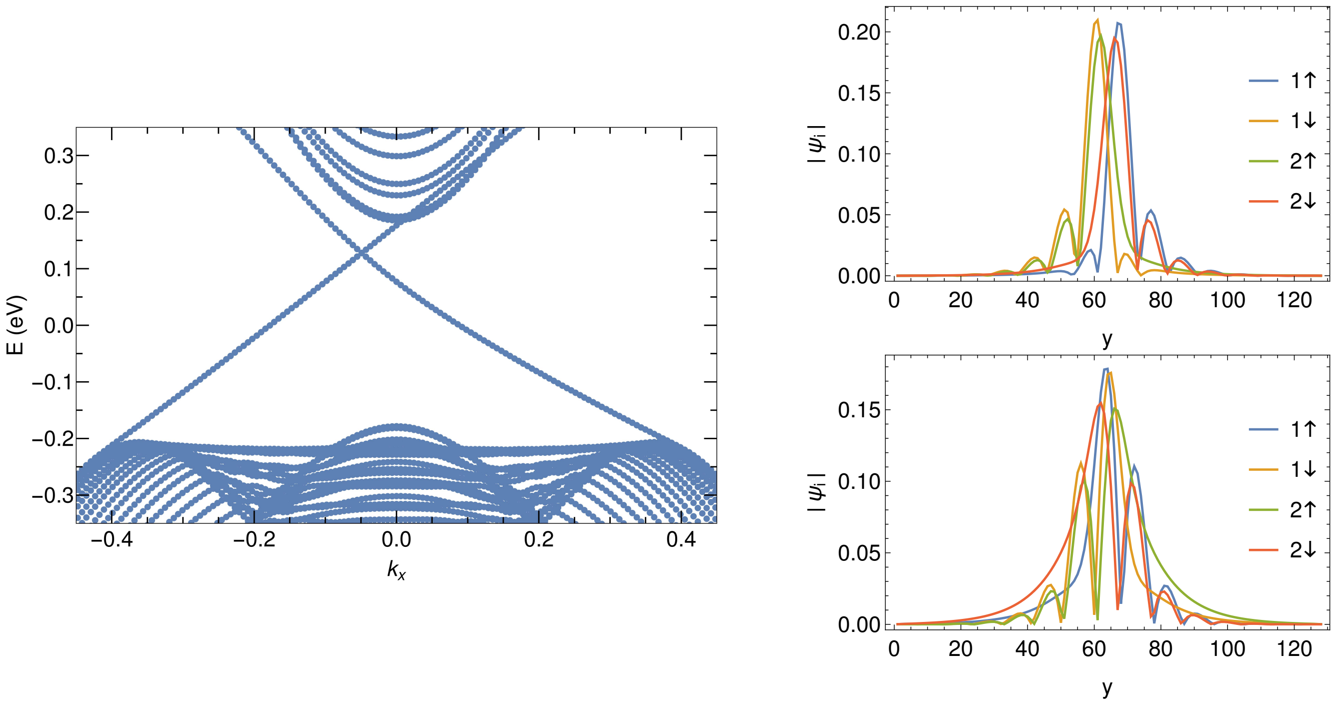

Figure S6. Numerical dispersion and eigenstates of an FTI stripe with domain wall in the center, bounded by vacuum in y-direction and with periodic boundary conditions in x-direction. The in-gap bands are twofold degenerate each, where those with positive slope belong to the edge states located at the FTI-vacuum interface. Bands with negative slope correspond to eigenstates located at the domain wall. On the right hand side the four components of these two eigenstates are shown for $k_x = 0$ illustrating their spin, orbital, and spatial structure. While edge states at the outer edge possess only contributions from two orbitals (like $1\uparrow$ and $2\downarrow$) resulting in a total z-polarization, eigenstates at the domain wall possess contributions from all orbitals.

Fig. S6, and therefore are not strictly z-polarized. Considering now the case shown in Fig. 2**d** of Ref. 1, this allows spin flip scattering at the points where the wave packet enters and leaves the domain wall structure. However, as spin-up and spin-down states are scattered symmetrically into the two exit channels, the sum of both leads to a perfect transmission through the device structure for almost the full energy range of the bulk gap (see Fig. S7). Back-scattering only occurs at the lower edge of the bulk gap, which increases the resistance of the device for such energies. Thus, the overall functionality of the spin transistor device remains unaffected by a domain wall structure.

References

1. Götte, M., Joppe, M. & Dahm, T. Pure spin current devices based on ferromagnetic topological insulators

2. Krueckl, V. & Richter, K. Switching spin and charge between edge states in topological insulator constrictions *Phys. Rev. Lett.* **107,** 086803 (2011).

3. Wakatsuki, R., Ezawa, M. & Nagaosa, N. Domain wall of a ferromagnet on a three-dimensional topological insulator *Sci. Rep.* **5,** 13638 (2015).

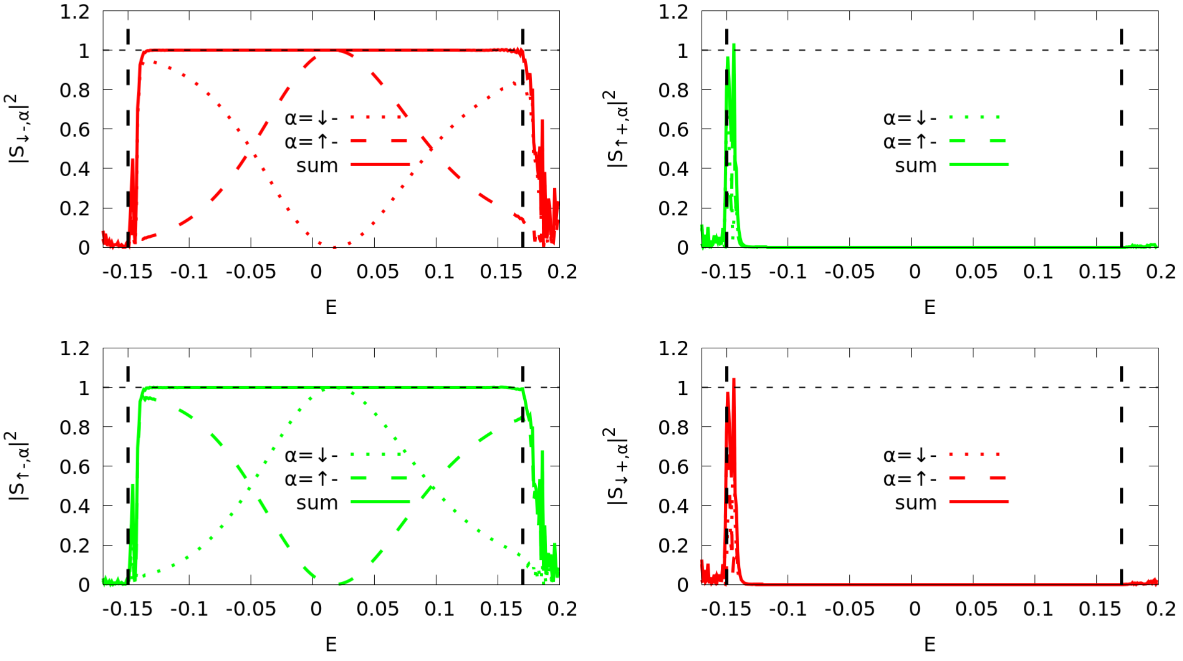

Figure S7. Scattering probabilities of the two wave packets Ψ_α starting on the right side of the device into the two exit channels $\Phi^-_\downarrow$ and $\Phi^-_\uparrow$ on the left (left panel). The sum of both scattering probabilities is equal for both exit channels and even equals 1 over the largest part of the bulk gap (indicated by the vertical black lines). Some back-scattering occurs only at the lower edge of the bulk gap (right panel).
